# The control processes and subjective well-being of Chinese teachers: evidence of convergence with and divergence from the key propositions of the motivational theory of life-span development

**DOI:** 10.3389/fpsyg.2014.00467

**Published:** 2014-05-21

**Authors:** Wan-chi Wong, Yin Li, Xiaoyan Sun, Huanu Xu

**Affiliations:** ^1^Department of Educational Psychology, The Chinese University of Hong KongChina; ^2^Department of Education and Human Development, Peking UniversityChina; ^3^Department of Psychology, Wuhan UniversityChina

**Keywords:** control processes, primary control, secondary control, subjective well-being, motivational theory of life-span development

## Abstract

An analytical review of the motivational theory of life-span development reveals that this theory has undergone a series of elegant theoretical integrations. Its claim to universality nonetheless brings forth unresolved controversies. With the purpose of scrutinizing the key propositions of this theory, an empirical study was designed to examine the control processes and subjective well-being of Chinese teachers (*N* = 637). The OPS-Scales (Optimization in Primary and Secondary Control Scales) for the Domain of Teaching were constructed to assess patterns of control processes. Three facets of subjective well-being were investigated with the Positive and Negative Affect Schedule, the Life Satisfaction Scale, and the Subjective Vitality Scale. The results revealed certain aspects of alignment with and certain divergences from the key propositions of the motivational theory of life-span development. Neither “primacy of primary control” nor “primacy of secondary control” was clearly supported. Notably, using different criteria for subjective well-being yielded different subtypes of primary and secondary control as predictors. The hypothesized life-span trajectories of primary and secondary control received limited support. To advance the theory in this area, we recommend incorporating Lakatos' ideas about sophisticated falsification by specifying the hard core of the motivational theory of life-span development and articulating new auxiliary hypotheses.

## Introduction

The motivational theory of life-span development, newly proposed by Heckhausen et al. in 2010, is by nature a reformulation and specification of the life-span theory of control, which was first formulated in the 1990s (Schulz et al., 1991; Heckhausen and Schulz, 1995). Substantiated by refined postulates on the role of self-regulation in human motivation in terms of primary and secondary control, the explication of the original and reformulated theory has garnered attention in the scientific community. With the purpose of scrutinizing the key propositions of this theory, an empirical study that focused on examining the control processes and subjective well-being of Chinese teachers was designed. Evidence of convergence with and divergence from the key propositions of the examined theory should provoke us to think dialectically between the theoretical and empirical levels and to find avenues for further theoretical development.

### The formulation of the motivational theory of life-span development through various waves of elegant integration

The conceptualization and empirical investigation of control processes has been an active area of research in recent decades (e.g., Rothbaum et al., 1982; Weisz, 1986; Holahan et al., 1996; Morling and Evered, 2006), and the proposal of the life-span theory of control (Heckhausen and Schulz, 1995) and related research programs (e.g., Heckhausen, 1997, 1999; Wrosch and Heckhausen, 1999; Wrosch et al., 2000) have served as landmarks. The life-span theory of control, which originated in the early 1990s (see Heckhausen and Schulz, 1990; Schulz et al., 1991), was subsequently specified with a model of optimization in primary and secondary control (OPS model; Heckhausen and Schulz, 1993, 1995). Immediately following these endeavors, the action-phase model of developmental regulation (Heckhausen, 1999, 2000) was formulated based on the Rubicon model of action phases (H. Heckhausen, 1987, 1989; H. Heckhausen and Gollwitzer, 1987). Each new formulation or specification represents a wave of elegant integration, culminating with the motivational theory of life-span development (Heckhausen et al., 2010). It is noteworthy that the 15 propositions of this newly formulated theory can be identified in one of the aforementioned waves of integration.

The life-span theory of control was built upon the existing conceptual and empirical resources of primary and secondary control in the early 1990s (see Schulz et al., 1991; Heckhausen and Schulz, 1995). Based on the distinction that primary control refers to an intervention within the environment, whereas secondary control refers to regulation by an internal process, the theory is characterized by two major propositions: the primacy of primary control and the life-span trajectories of primary and secondary control. The hypothesis that primary control has primacy means that this type of control “is both preferred and has a greater adaptive value to the individual” (Heckhausen and Schulz, 1995, p. 286). In terms of the life-span trajectories of developmental regulation, it is hypothesized that striving for primary control remains stable throughout adult life course, whereas striving for secondary control increases beginning in childhood through midlife until old age. In terms of primary control capacity, an inverted U-shape over the life-span is hypothesized.

Life-span theory also assumes that selectivity and compensation are fundamental requirements of human behavior and development (Heckhausen and Schulz, 1993, 1995). Selectivity (i.e., the selection of goals and focused investment of resources in goal attainment) is needed to achieve successful behavior-event contingencies. In contrast, compensation is needed to protect individuals' motivational resources when facing experiences of failure, loss, threat, and decline, all of which are inevitable and frequent in life. By integrating these two fundamental requirements with the conceptual distinction between primary and secondary control, four types of control strategies are identified: (1) selective primary control (SPC) (e.g., investing effort and time, learning new skills), (2) compensatory primary control (CPC) (e.g., seeking the advice and help of others, using technical aids), (3) selective secondary control (SSC) (e.g., enhancing goal value, anticipating the positive consequences of goal attainment), and (4) compensatory secondary control (CSC) (e.g., disengaging from goals, making self-protective interpretations). These categories serve as important conceptual tools in subsequent empirical inquires.

It is further postulated that the effective use of the four types of control strategies is regulated by a higher-order process known as optimization, which serves as the regulatory mechanism for selection and compensation that optimizes the long-term potential of primary control (Heckhausen and Schulz, 1993). This specification of the life-span theory is also known as the Model of Optimization in Primary and Secondary Control (OPS model), which can be perceived as an expansion of Baltes and Baltes' (1990) model of selective optimization with compensation. The major contribution of the OPS model lies in identifying the heuristics of adaptive goal choices for optimizing development, which are succinctly summarized as matching goals to opportunities, managing interdomain and long-term consequences, and maintaining the diversity of goals (Heckhausen et al., 2010). The general OPS-Scales (Heckhausen et al., 1998), which is a multi-scale questionnaire, was developed and applied in related research programs.

The formulation of the action-phase model of developmental regulation (Heckhausen, 1999, 2000) is an extension of the Rubicon model (H. Heckhausen, 1987, 1989; H. Heckhausen and Gollwitzer, 1987) in two important ways: (1) it specifies the employment of control strategies in each phase of the motivational process, and (2) it supplements the concept of a “developmental deadline” as an additional Rubicon in the phase model of human motivation. In this extended model, goal engagement and goal disengagement are conceptualized as two distinct states. The key postulations of the model are as follows: goal engagement involves SPC, SSC, and CPC, which intensifies immediately before a “deadline” situation (characterized by diminishing opportunities for goal attainment); goal disengagement, which is caused by a failure or a setback, typically involves CSC, which would be adaptive in such circumstances. In this model, it is evident that SSC is postulated as the preferred and adaptive strategy for goal engagement. How to reconcile this proposition with the key proposition of the primacy of primary control is a theoretical issue that has not yet been properly addressed.

The proposal of the motivational theory of life-span development represents a conscious attempt by Heckhausen et al. (2010) to merge the OPS model and the phase model of developmental regulation with the original life-span theory of control. Under this new umbrella term, Heckhausen and her associates formulated 15 propositions within the following four areas: (1) the primacy of primary control, (2) the life-span trajectories of primary and secondary control, (3) the optimization of control choice and the use of control strategies, and (4) the action phases of goal choice, goal engagement, goal disengagement, and goal reengagement. While these propositions were not new, Heckhausen and her associates brought them together in an organized scheme and supplemented them with updated empirical evidence. Notably, the key propositions of the original life-span theory of control remain the cornerstone of the newly formulated motivational theory of life-span development.

### The longstanding debate regarding the cultural differences in primary and secondary control strivings

The debate over cultural differences in primary and secondary control strivings can be traced back to the 1980s (see Azuma, 1984; Weisz et al., 1984). In formulating the life-span theory of control, Heckhausen and Schulz (1993, 1995) devised a relatively refined conceptualization of secondary control and primary control, even though the primacy of primary control serves as a key proposition. The theory's claim of universality aroused a heated debate that was initiated by Gould (1999). Drawing on empirical studies conducted in Asian societies, Gould suggested that secondary control has primacy in that part of the world. Heckhausen and Schulz's theoretical explication was criticized by Gould as another example of the “imposed etic” perspective, which commits the typical error of interpreting non-Western cultures through a Western lens. In their reply to Gould's critique, Heckhausen and Schulz (1999) made the following two essential clarifications: (1) the fundamental characteristics of the human motivation system, which constitute the heart of the life-span theory of control, are proposed to be historically and culturally invariant, and (2) secondary control does not play the role of a master or a slave in relation to primary control. Instead, the metaphor for the relationship of secondary control to primary control is that of a confederate. Attempts to substantiate the functional primacy of primary control across time and cultures were completed by drawing support from theoretical perspectives and empirical evidence from evolutionary, comparative, developmental, and cultural psychology (Heckhausen and Schulz, 1999).

A closer examination of Gould's (1999) perspective reveals that his interpretation is limited by a stereotypical understanding of Asian cultures and people. His contention of the primacy of secondary control in Asian cultures was inferred from findings related to the collectivistic orientation and/or interdependent self of the Asian population. Only a limited number of studies reviewed by Gould (1999) directly measured primary control and/or secondary control in Asian societies. A large-scale study conducted by Wong et al. (2006) that included mainland Chinese students served to fill this gap. This study found that SPC, SSC, and CPC were extensively employed in academic pursuits across different situations. In situations of failure, primary control and SSC (but not compensatory secondary control) were found to be adaptive. These results do not lend support to the primacy of secondary control in Asian societies.

Joining the discourse on the primacy of primary control or secondary control, Yamaguchi (2001) noted that empirical research on this issue is scarce. According to his review of the existing literature, the claim that East Asians use less primary control compared with Westerners is not well supported. Nonetheless, on the basis of his conceptual analysis, he suggested that secondary control could contribute to psychological well-being among East Asians. In his new conceptualization, the primacy of primary or secondary control depends on the urgency of biological needs and the dominant cultural values of autonomy vs. the maintenance of harmony.

The debate over the cultural differences in primary and secondary control gained momentum with Morling and Evered's (2006) comprehensive review on secondary control, which directed special attention at compiling the paraphrased and operationalized definitions of the construct. In defining “adjustment of the self” and “acceptance of the environment” as two key aspects of secondary control, they further advocated for a fit-focused definition of the construct that incorporated both aspects. Notably, Morling and Evered (2006) categorized the conceptualization of Heckhausen and her associates as a control-focused definition of secondary control that was concerned solely with the adjustment of the self. Careful deliberation leads us, nonetheless, to the question of whether “adjustment of the self” and “acceptance of the environment” can be considered independent of each other in the context of applying a certain secondary control strategy. In their conceptualization of SSC, it is evident that Heckhausen and Schulz (1993, 1995) did not consider a fit-focused orientation, thus widening the scope of secondary control. A closer examination of the prototypical content of CSC further reveals that the adjustment of self is related to the acceptance of the current situation in a subtle way, regardless of whether coping is used for goal disengagement or as a self-protective mechanism.

A noteworthy contribution of Morling and Evered's (2006) review article is the proposal of a multiple-motive view of functionality with regard to control striving. While a sense of agency is considered to be the major underlying motive in primary control striving, fit-focused secondary control could be related to various motives, such as a longing for relatedness, serenity, coherence, or the meaning of life. Based on this analysis, Morling and Evered criticized Heckhausen and Schulz's research paradigm for its limited consideration of human motives. Expanding on this understanding, they also perceived the adaptive values of fit-focused secondary control to be logical consequences of the fulfillment of basic needs.

Based on their review of the empirical evidence organized around the fit-focused definition, Morling and Evered (2006) claimed that secondary control is often associated with positive outcomes. Furthermore, they asserted that secondary control was found to be more relevant, elaborated, and preferred in non-Western cultures. This review clearly diverges from the review by Heckhausen and her associates (Heckhausen et al., 2010), which offered evidence supporting the primacy of primary control in both Western and Eastern cultures. When considering the different conclusions of these reviews, it is important to note that the conceptualization and the measurement of secondary control differ in different research studies.

### Subjective well-being as an area of active research in recent decades

Broadly defined, subjective well-being refers to the good life in the classical Greek sense. Contemporarily, it is sometimes used as a synonym for the concept of happiness. Given its central place in human life, it is not surprising that subjective well-being has become an active area of research in recent decades. Highlighting the subjective aspect, this construct refers to a psychological state during which “a person feels and thinks that his or her life is desirable regardless of how others see it” (Diener, 2009, p. 1).

In his seminal review of subjective well-being, Diener (1984) distinguished bottom-up and top-down processes that influence subjective well-being. Bottom-up processes include external impacts (e.g., circumstantial and demographic factors), whereas top-down processes refer to the impacts of psychological factors. In a more updated review entitled “Subjective well-being: Three decades of progress,” Diener et al. (1999) made an important clarification: Because circumstantial and demographic factors account for a small amount of the variance in subjective well-being, researchers have turned to psychological factors to explain the variability. Among the psychological factors, goal regulation and coping efforts have been identified as important influences on well-being.

In addition to the efforts to explain the factors that affect subjective well-being, serious attention has been devoted to the adequate conceptualization and measurement of the construct. “Global life satisfaction” and “a preponderance of positive affect over negative affect” have been identified as key components of subjective well-being for decades (see Diener, 2009). Ryan and Frederick (1997) further highlighted subjective vitality as a dynamic reflection of well-being. When studying the adaptive values of control strategies, Heckhausen and her associates (e.g., Wrosch and Heckhausen, 1999; Heckhausen et al., 2001) applied the construct of subjective well-being and measured it with the Positive and Negative Affect Schedule (PANAS). To capture the facets of subjective well-being in a more comprehensive way, it is recommended that the Satisfaction with Life Scale and the Subjective Vitality Scale be used in addition to the PANAS.

### The present study

Theoretical advancements in control processes offer rich possibilities for generating empirical studies on diverse domains of human life. In light of the current state of knowledge and the debate concerning the universality of the motivational theory of life-span development, the present study aimed to investigate the patterns of control processes and subjective well-being and their relationship among Chinese teachers. By recruiting an East Asian sample with a large age range (20–60 years), the present study allowed for the empirical scrutiny of the following three key propositions of the motivational theory of life-span development: (1) primary control striving is preferred; (2) primary control striving has benefits; and (3) primary control striving is stable, whereas secondary control striving increases across adulthood (see Heckhausen et al., 2010, p. 42). Propositions one and two constitute the “primacy of primary control” thesis, and proposition three is concerned with “life-span trajectories of primary and secondary control.”

Based on the framework of the motivational theory of life-span development, three predictions were made in the present study: (1) Chinese teachers frequently endorse primary control in their everyday teaching practices; (2) primary control striving predicts subjective well-being in Chinese teachers; and (3) there are no significant differences in primary control striving between Chinese teachers of different ages, but a stronger tendency toward using secondary control is observed in Chinese teachers of more advanced ages. Evidence that converged with or diverged from the three examined key propositions of the motivational theory of life-span development was carefully deliberated. The significance of the present study lies in its potential contribution to the theoretical advancement of control processes. The results can also lead to a more refined understanding of how coping efforts affect subjective well-being, which has both theoretical and practical implications.

## Methods

### Participants and procedure

The sample of teachers (*N* = 637, 58% female teachers) was drawn from secondary schools in the Guangdong and Hebei provinces, which are located in the southern and northern parts of mainland China, respectively. The teachers varied in age (range = 20–60 years, *M* = 34.68, *SD* = 7.30) and educational qualifications (diploma, undergraduate, and postgraduate), but they all worked in schools of average academic standards in one of three cities (two in Guangdong and one in Hebei). All teachers participated voluntarily; they responded to an invitation to participate in a teacher development project. Along with being invited to respond to a questionnaire about control processes, they were also asked to complete three scales reflecting subjective well-being. They participated in these tasks during their free time or during a teacher development course, and there were no time constraints.

### Instruments

#### The OPST (OPS-scales for the domain of teaching)

The OPS-Scales for the Domain of Teaching (OPST) was adapted from the general OPS-Scales (Heckhausen et al., 1998) and the OPSAA (OPS-Scales for the domain of Academic Achievement; see Wong et al., 2006), which underwent a back-translation process. The original OPSAA was composed of six subscales (general optimization, optimization in the domain of academic achievement, selective primary control, selective secondary control, compensatory primary control, and compensatory secondary control) that each contained five items. Based on the results of factor analysis carried out in a previous study among mainland Chinese students (Wong et al., 2006), we divided the compensatory primary control (CPC) and compensatory secondary control (CSC) subscales into more specific subcategories (CPCa, CPCb, CSCa, CSCb) and constructed new items according to the meaning of the new subcategories. Each new subcategory contained five items.

After carefully constructing the items, we used the 40-item OPST in a pilot study among secondary school teachers in the Guangdong Province of mainland China (*N* = 204). Each item portrays a type of optimization or control strategy evidenced in an act or a thought. The respondents were instructed to review their own state and to report the frequency of the occurrence of the stated strategies in their everyday teaching life using a 5-point Likert scale (1 = *never true* to 5 = *almost always true*).

Based on the item analysis of the OPST subscales, we made minor revisions to three items that belonged to the general optimization subscale, the selective secondary control subscale, and the compensatory secondary control subscale, respectively. The revised OPST used in the present study consisted of 40 items (five items × eight subscales). Excluding the 10 items on optimization, 30 items were related to control strategies (five items × six subscales). Table 1 shows the prototypical item content of the OPST subscales and the reliabilities (αs: standardized alpha) of these subscales (ranging from 0.67 to 0.84) that were revealed in the main study.

**Table 1:** **The prototypical item content of the OPST control subscales and optimization subscales**.

**(1) Selective primary control (SPC)**
Prototypical item content
Investing effort, time, and/or energy in the attainment of teaching goals; developing essential skills and abilities to achieve a teaching goal; fighting difficulties in the realization of a teaching goal
α = 0.80
**(2) Compensatory primary control (Type a) (CPCa)**
Prototypical item content
Seeking help or advice from others in different teaching situations
α = 0.84
**(3) Compensatory primary control (Type b) (CPCb)**
Prototypical item content
Trying new, unfamiliar, or unusual ways of overcoming setbacks in teaching practices; taking note of others' methods; learning more effective ways of solving difficulties in the teaching practices
α = 0.76
**(4) Selective secondary control (SSC)**
Prototypical item content
Enhancing the value of teaching goals or devaluing competing goals; enhancing perception of control in a teaching goal; anticipating the positive consequences of attaining the teaching goal
α = 0.75
**(5) Compensatory secondary control (Type a) (CSCa)**
Prototypical item content
Self-protective interpretations for setbacks in teaching life: external attribution, self-protective social comparison, and self-protective intra-individualized comparison
α = 0.78
**(6) Compensatory secondary control (Type b) (CSCb)**
Prototypical item content
Goal disengagement or goal adjustment following a failure in teaching practices
α = 0.76
**(7) General optimization (OG)**
Prototypical item content
Maintenance of diversity in the general domain of human life; management of positive and negative trade-offs for other life domains and future life course
α = 0.67
**(8) Optimization in the domain of teaching (OT)**
Prototypical item content
Adaptive goal selection and maintenance of diversity in the domain of teaching
α = 0.75

α = standardized Cronbach's alpha.

To capture the facets of subjective well-being in a more comprehensive way, three scales were applied: the PANAS, the Satisfaction with Life Scale, and the Subjective Vitality Scale.

#### The PANAS (positive and negative affect schedule)

The 20-item PANAS, a widely applied instrument for assessing subjective well-being, was constructed by Watson et al. (1988). The PANAS has demonstrated good psychometric properties, including high inter-item reliability scores, high convergences in factor structures, and high discriminant validity (Watson, 1988; Watson et al., 1988). The PANAS consists of two 10-item (each in the form of an adjective) self-report scales designed to assess positive and negative affect. Specifically, the 10 positive affect items are “interested,” “excited,” “strong,” “enthusiastic,” “proud,” “alert,” “inspired,” “determined,” “attentive,” and “active.” The negative affect items are “distressed,” “upset,” “guilty,” “scared,” “hostile,” “irritable,” “ashamed,” “nervous,” “jittery,” and “afraid.” The present study adopted the Chinese version that was developed by Wong and Li (see Wong et al., 2006), which underwent a back-translation process. Based on the item analysis of a pilot study involving secondary school teachers in Guangdong Province (*N* = 189), slight revisions were made to two items to improve their clarity in conveying the equivalent meaning of the original items, namely, “alert” and “irritable.” In the present study, the subjects were instructed to report how frequently they experienced positive and negative affect in their everyday teaching life using a 5-point Likert scale (1 = *very slightly or not at all* to 5 = *extremely*). The standardized alpha values of the positive affect subscale and negative affect subscale were 0.87 and 0.85, respectively.

#### The Satisfaction with Life Scale

Diener et al. (1985) intended to develop a scale to measure subjective well-being by highlighting a global sense of life satisfaction. A five-item scale was developed from 10 items that loaded onto a factor interpreted as life satisfaction, which was one of three factors that emerged from a pool of 48 items. The other two factors were positive affect and negative affect. The five items on the Satisfaction with Life Scale are as follows: (1) “In most ways my life is close to my ideal,” (2) “The conditions of my life are excellent,” (3) “I am satisfied with my life,” (4) “So far I have gotten the important things I want in life,” and (5) “If I could live my life over, I would change almost nothing.” In a review of the psychometric properties of this five-item scale, Pavot and Diener (1993) reported that it possessed good convergent validity and showed discriminant validity from an emotional well-being measure. In the present study, the subjects were instructed to use a 6-point Likert scale (1 = *strongly disagree* to 6 = *strongly agree*) to respond to the Satisfaction with Life Scale with respect to their current teaching life. The scale underwent a back-translation process and was piloted on secondary school teachers (*N* = 189) in Guangdong Province of mainland China. Following the item analysis of the scale in the pilot study, the wording of one item was slightly revised to improve its clarity in conveying the equivalent meaning of the original item. The standardized alpha of the Satisfaction with Life Scale in the present study was 0.77.

#### The subjective vitality scale

Ryan and Frederick (1997) developed a measure of subjective vitality as a reflection of well-being. In the initial phase, 19 items were constructed. In a study of 190 subjects, seven items loaded onto the first factor (eigenvalue = 6.77, α = 0.84), clearly indicating a state of vitality and energy. In two additional studies that involved 190 and 376 subjects, respectively, the internal consistency of this seven-item scale was good (α = 0.84 and 0.86). The seven items on this scale are as follows: (1) “I feel alive and vital,” (2) “I don't feel very energetic (reverse-scored item),” (3) “Sometimes I feel so alive I just want to burst,” (4) “I have energy and spirit,” (5) “I look forward to each new day,” (6) “I nearly always feel alert and awake,” and (7) “I feel energized.” In the present study, the subjects were instructed to use a 6-point Likert scale (1 = *strongly disagree* to 6 = *strongly agree*) to respond to the Subjective Vitality Scale with respect to their current teaching life. The scale underwent a back-translation process and was then piloted in a study that involved 189 secondary school teachers in Guangdong Province of mainland China. The standardized alpha of the Subjective Vitality Scale in the present study was 0.84.

### Analysis

Three sets of analyses, corresponding to the three major predictions of the present study, were conducted. To address the question concerning the frequency of endorsing primary vs. secondary control, we compared the mean scores for each type of control strategies by performing One-Way repeated measures ANOVA. Next, we conducted a series of hierarchical multiple regression analyses to explore the effects of varied control strategies on the different facets of subjective well-being, including positive and negative affect, satisfaction with life, and subjective vitality. Finally, we conducted One-Way ANOVAs to compare the primary and secondary control strivings among Chinese teachers in different age groups.

## Results and discussion

This section is organized based on the underlying analyses of the three predictions of the present study. As a precedent step, we report the results of a confirmatory factor analysis (CFA) that indicated the structural validity of the OPST, which was the key measure in this study. We examined the hypothesized eight-factor structure of OPST (six factors on control strategies: SPC, CPCa, CPCb, SSC, CSCa, and CSCb; two factors on optimization: OG and OT) by performing a CFA with Mplus 6.0 (Muthén and Muthén, 2010). Fit indices obtained help to determine how well the hypothesized model fits the sample data. When reporting fit indices, Kline (1998/2011) recommends including the chi-square test, CFI, RMSEA, and SRMR. In the CFA of the hypothesized eight-factor structure of the OPST, we obtained the following results: χ^2^ = 1821.62, *df* = 712, *p* < 0.001, CFI = 0.88, RMSEA = 0.055, the 90% confidence interval for RMSEA = (0.052; 0.058), SRMR = 0.06. Based on Hu and Bentler's (1999) recommendation of the combinational rule that specifies the cutoff criteria for the RMSEA and SRMR fit indices (RMSEA ≤ 0.06 and SRMR ≤ 0.09), the hypothesized model fits the observed data well, and one can have reasonable confidence about the goodness of fit of the model. According to Hu and Bentler (1999), using this combinational rule is more preferable for model evaluation because it results in the least sum of Type I and Type II error rates.

### The endorsement of primary and secondary control strategies among the chinese teachers

The OPST was designed to elicit how frequently the teachers endorsed the different types of control strategies during everyday teaching practices. Table 2 summarizes the mean scores and *SD*s of the OPST subscales that reflect the patterns of control processes among Chinese teachers.

**Table 2:** **Means and *SD*s of the OPST control subscales and optimization subscales (*N* = 637)**.

	**Mean**	**Mean per item**	***SD***
**OPST CONTROL SUBSCALES**			
SPC	20.00	4.00	3.72
CPCa	19.25	3.85	4.00
CPCb	18.73	3.75	3.60
SSC	18.98	3.80	3.59
CSCa	14.66	2.93	4.02
CSCb	15.42	3.08	3.89
**OPST OPTIMIZATION SUBSCALES**			
OG	17.80	3.56	3.60
OT	18.80	3.76	3.67

The SDs were calculated from the sums of the subscales.

SPC, selective primary control; CPCa, compensatory primary control (Type a); CPCb, compensatory primary control (Type b); SSC, selective secondary control; CSCa, compensatory secondary control (Type a); CSCb, compensatory secondary control (Type b); OG, general optimization; OT, optimization in the domain of teaching.

From the mean scores shown in Table 2, it is evident that both selective primary control (SPC) and compensatory primary control (CPC) were preferred. A much lower mean score for compensatory secondary control (CSC) demonstrates that this type of strategy was employed less frequently in this sample of Chinese teachers. Nonetheless, it should be noted that selective secondary control (SSC) was also a frequently employed strategy following selective primary control and compensatory primary control (Type a).

To test whether the mean endorsement of the different types of control strategies significantly differs from one another, we submitted those means to One-Way repeated measures ANOVA, with the type of control strategies as within-subject variable. The results confirmed that there was significant difference in the application of the six control strategies, *F*_(5, 632)_ = 162.40, *p* < 0.001, η^2^_*p*_ = 0.56. In accordance with this, Bonferroni-corrected comparisons further located a series of significant difference in the endorsement of these control strategies. Except the differences between SSC vs. CPCa (*p* = 0.10) and between SSC vs. CPCb (*p* = 1.00), other pairwise comparisons among the six control strategies were significant (*p*s < 0.001).

Although the data indicate that primary control was preferred, we should not ignore the fact that selective secondary control was also frequently endorsed. It is worthy to note that there is no significant difference between the mean endorsement of selective secondary control and the two subtypes of compensatory primary control. Taken together, the Chinese teachers preferred control strategies that fell into the category of goal engagement over those that involved, by nature, disengagement from teaching goals. In addition, it was observed that Chinese teachers quite heavily endorsed optimization strategies in the domain of teaching (OT).

### The predictors of subjective well-being among chinese teachers

To address our research question about the primacy of primary control, it is necessary to further explore the adaptive values or benefits of primary control. The focus of the present study was to examine whether the various types of primary and secondary control are significant predictors of subjective well-being among teachers. To capture subjective well-being in a more comprehensive way, various criteria were adopted, including positive and negative affect, life satisfaction, and subjective vitality. Tables 3A,B shows the bivariate correlations between the study variables, which serve as the preliminary bases of a more rigorous procedure of the hierarchical multiple regression analyses.

**Table 3A:** **Bivariate correlations between the demographic variables and control processes**.

	**1**	**2**	**3**	**4**	**5**	**6**	**7**	**8**	**9**	**10**	**11**	**12**
1. Sex	–											
2. Age	−0.15^**^	–										
3. Position	−0.06	0.38^***^	–									
4. Grade level taught	−0.09^*^	0.16^***^	0.14^**^	–								
5. Qualification	−0.01	−0.09^*^	−0.02	0.10^*^	–							
6. OG	0.00	−0.01	−0.03	−0.21^***^	0.04	–						
7. OT	0.02	−0.07	−0.09^*^	−0.27^***^	0.06	0.67^***^	–					
8. SPC	0.07	0.03	−0.02	−0.22^***^	0.01	0.51^***^	0.76^***^	–				
9. CPCa	0.16^***^	−0.15^**^	−0.07	−0.22^***^	0.04	0.57^***^	0.63^***^	0.65^***^	–			
10. CPCb	0.02	0.00	−0.06	−0.22^***^	0.06	0.66^***^	0.77^***^	0.75^***^	0.67^***^	–		
11. SSC	−0.03	0.02	0.00	−0.20^***^	0.04	0.63^***^	0.76^***^	0.73^***^	0.61^***^	0.72^***^	–	
12. CSCa	−0.03	0.02	0.01	−0.19^***^	0.06	0.48^***^	0.33^***^	0.13^**^	0.27^***^	0.33^***^	0.28^***^	–
13. CSCb	−0.04	0.00	−0.01	−0.24^***^	0.03	0.58^***^	0.43^***^	0.20^***^	0.34^***^	0.41^***^	0.37^***^	0.77^***^

* p < 0.05;

** p < 0.01;

*** p < 0.001.

OG, general optimization; OT, optimization in the domain of teaching; SPC, selective primary control; CPCa, compensatory primary control (Type a); CPCb, compensatory primary control (Type b); SSC, selective secondary control; CSCa, compensatory secondary control (Type a); CSCb, compensatory secondary control (Type b).

**Table 3B:** **Bivariate correlations between the facets of subjective well-being and the major study variables**.

	**Positive affect**	**Negative affect**	**Satisfaction in teaching life**	**Subjective vitality**
1. Sex	0.01	−0.04	0.01	−0.05
2. Age	−0.02	0.02	0.08	0.00
3. Position	−0.03	−0.06	0.04	−0.02
4. Grade level taught	−0.05	0.12^**^	−0.22^***^	−0.24^***^
5. Qualification	0.02	−0.06	0.02	0.01
6. OG	0.30^***^	−0.27^***^	0.37^***^	0.47^***^
7. OT	0.46^***^	−0.29^***^	0.37^***^	0.54^***^
8. SPC	0.49^***^	−0.26^***^	0.30^***^	0.49^***^
9. CPCa	0.30^***^	−0.22^***^	0.30^***^	0.37^***^
10. CPCb	0.44^***^	−0.28^***^	0.37^***^	0.50^***^
11. SSC	0.44^***^	−0.26^***^	0.38^***^	0.50^***^
12. CSCa	−0.03	−0.06	0.36^***^	0.18^***^
13. CSCb	0.08	−0.14^**^	0.40^***^	0.26^***^

** p < 0.01;

*** p < 0.001.

OG, general optimization; OT, optimization in the domain of teaching; SPC, selective primary control; CPCa, compensatory primary control (Type a); CPCb, compensatory primary control (Type b); SSC, selective secondary control; CSCa, compensatory secondary control (Type a); CSCb, compensatory secondary control (Type b).

To examine the effects of control processes on individuals' subjective well-being, a series of hierarchical regression analyses was conducted. In the first step of each regression analysis, five demographic variables (age, sex, position, grade level taught, and qualification) were entered to control for their possible covariate effects on a certain criterion of subjective well-being. In the second step, the eight subscales of the OPST (including six control strategy variables and two optimization variables) were entered as one block.

### Hierarchical multiple regression analyses with positive and negative affect as the criteria and control processes as predictors

The construct “positive affect” has always been recognized as an important facet of subjective well-being. Table 4 shows the results of a hierarchical regression analysis with positive affect as the criterion.

**Table 4:** **Summary of hierarchical multiple regression analysis predicting positive affect from demographic variables and control processes**.

	**Predictor**	***R*^2^**	**Δ*R*^2^**	***B***	***SE***	β
**Step 1**		0.004	0.004			
	Intercept			34.28^***^	2.75	
	Age			0.02	0.05	−0.00
	Sex			−0.03	0.59	−0.00
	Position			−0.14	0.41	−0.02
	Grade level taught			−0.76	0.60	−0.06
	Qualification			0.62	0.87	0.03
**Step 2**		0.29^***^	0.29^***^			
	SPC			0.45	0.14	0.23^**^
	SSC			0.21	0.14	0.11
	CPCa			−0.23	0.10	−0.13^*^
	CPCb			0.30	0.14	0.15^*^
	CSCa			−0.36	0.10	−0.21^***^
	CSCb			0.09	0.11	0.05
	OG			0.08	0.11	0.04
	OT			0.38	0.14	0.20^**^

* p < 0.05;

** p < 0.01;

*** p < 0.001.

SPC, selective primary control; SSC, selective secondary control; CPCa, compensatory primary control (Type a); CPCb, compensatory primary control (Type b); CSCa, compensatory secondary control (Type a); CSCb, compensatory secondary control (Type b); OG, general optimization; OT, optimization in the domain of teaching.

The results show that demographic variables explained only 0.4% of the variance in the positive affect score [*R*^2^ = 0.004, *F*_(5, 560)_ = 0.44, *ns*.]. Among the control strategy and optimization variables, SPC, CPCa, CPCb, CSCa, and OT were significant predictors of the positive affect score after controlling for the demographic predictors (βs = 0.23, −0.13, 0.15, −0.21, and 0.20, respectively, *p*s < 0.05). Together, these predictors explained an additional 29% of the variance in the positive affect score beyond the demographic predictors [Δ*R*^2^ = 0.29, Δ*F*_(8, 552)_ = 28.20, *p* < 0.001]. These results imply that optimization in teaching, selective primary control, and compensatory primary control (characterized by trying new and efficient ways of teaching) have adaptive values or benefits. It is not surprising that the CSCa subscale score (characterized by self-protective interpretations) negatively predicted the positive affect score. However, it is interesting to note that the CPCa subscale score (characterized by a readiness to seek help) also negatively predicted the positive affect score.

One way of defining subjective well-being is the “preponderance of positive affect over negative affect” (see Diener, 2009, p. 13). Thus, it is interesting to examine how control processes predict negative affect. Table 5 shows the results of a hierarchical regression analysis with negative affect as the criterion.

**Table 5:** **Summary of hierarchical multiple regression analysis predicting negative affect from demographic variables and control processes**.

	**Predictor**	***R*^2^**	**Δ*R*^2^**	***B***	***SE***	**β**
**Step 1**		0.03^**^	0.03^**^			
	Intercept			22.55^***^	2.55	
	Age			0.02	0.04	0.02
	Sex			−0.28	0.55	−0.02
	Position			−0.78	0.38	−0.09^*^
	Grade level taught			1.73	0.56	0.13^**^
	Qualification			−1.29	0.81	−0.07
**Step 2**		0.13^***^	0.11^***^			
	SPC			−0.02	0.14	−0.01
	SSC			−0.07	0.13	−0.04
	CPCa			0.03	0.10	0.02
	CPCb			−0.22	0.14	−0.12
	CSCa			0.22	0.10	0.14^*^
	CSCb			−0.06	0.12	−0.04
	OG			−0.25	0.12	−0.14^*^
	OT			−0.22	0.14	−0.12

* p < 0.05;

** p < 0.01;

*** p < 0.001.

SPC, selective primary control; SSC, selective secondary control; CPCa, compensatory primary control (Type a); CPCb, compensatory primary control (Type b); CSCa, compensatory secondary control (Type a); CSCb, compensatory secondary control (Type b); OG, general optimization; OT, optimization in the domain of teaching.

The results show that demographic variables explained 3% of the variance in the negative affect score [*R*^2^ = 0.03, *F*_(5, 559)_ = 3.03, *p* = 0.01]. Among the control strategy and optimization variables, only CSCa and OG were significant predictors of the negative affect score after controlling for demographic variables (βs = 0.14 and −0.14, respectively, *p*s < 0.05); CSCa positively predicted negative affect, and OG negatively predicted negative affect. Together, these predictors explained an additional 11% of the variance in the negative affect score beyond the demographic predictors [Δ*R*^2^ = 0.11, Δ*F*_(8, 551)_ = 8.45, *p* < 0.001]. These results imply that teachers who employ general optimization strategies less frequently and employ a form of compensatory secondary control that is characterized as self-protective more frequently are most apt to experience negative affect.

### Hierarchical multiple regression analysis with life satisfaction as the criterion and control processes as predictors

A global sense of life satisfaction has also been highlighted as a facet of subjective well-being. Table 6 shows the results of a hierarchical regression analysis with satisfaction in teaching life as the criterion.

**Table 6:** **Summary of hierarchical multiple regression analysis predicting satisfaction in teaching life from demographic variables and control processes**.

	**Predictor**	***R*^2^**	**Δ*R*^2^**	***B***	***SE***	β
**Step 1**		0.06^***^	0.06^***^			
	Intercept			17.67^***^	1.90	
	Age			0.07	0.03	0.10^*^
	Sex			−0.03	0.41	−0.00
	Position			0.22	0.29	0.04
	Grade level taught			−2.26	0.42	−0.23^***^
	Qualification			0.69	0.59	0.05
**Step 2**		0.25^***^	0.19^***^			
	SPC			−0.01	0.10	−0.01
	SSC			0.21	0.09	0.15^*^
	CPCa			0.02	0.07	0.01
	CPCb			0.11	0.10	0.08
	CSCa			0.12	0.07	0.10
	CSCb			0.21	0.08	0.17^*^
	OG			−0.01	0.08	−0.00
	OT			0.09	0.10	0.06

* p < 0.05;

*** p < 0.001.

SPC, selective primary control; SSC, selective secondary control; CPCa, compensatory primary control (Type a); CPCb, compensatory primary control (Type b); CSCa, compensatory secondary control (Type a); CSCb, compensatory secondary control (Type b); OG, general optimization; OT, optimization in the domain of teaching.

The results show that demographic variables explained 6% of the variance in the Satisfaction with Life score [*R*^2^ = 0.06, *F*_(5, 534)_ = 6.67, *p* < 0.001]. Among the control strategy and optimization variables, only SSC and CSCb were significant predictors of the Satisfaction with Life score after controlling for demographic variables (βs = 0.15 and 0.17, respectively, *p*s < 0.05). Together, these predictors explained an additional 19% of the variance within the Satisfaction with Life score beyond the demographic predictors [Δ*R*^2^ = 0.19, Δ*F*_(8, 526)_ = 16.27, *p* < 0.001]. These results suggest that two conditions are favorable for teachers' sense of satisfaction with their teaching lives: (1) more frequent endorsement of selective secondary control and (2) more frequent endorsement of compensatory secondary control that is characterized by goal disengagement or adjustment following failure in teaching practices. Notably, these results do not support the hypothesis of the primacy of primary control, which emphasizes the adaptive value of primary control.

### Hierarchical multiple regression analysis with subjective vitality as the criterion and control processes as predictors

Subjective vitality is another meaningful facet of subjective well-being; thus, a hierarchical multiple regression analysis was conducted with subjective vitality as the criterion, and the results are shown in Table 7.

**Table 7:** **Summary of hierarchical multiple regression analysis predicting subjective vitality from demographic variables and control processes**.

	**Predictor**	***R*^2^**	**Δ*R*^2^**	***B***	***SE***	**β**
**Step 1**		0.06^***^	0.06^***^			
	Intercept			33.56^***^	2.69	
	Age			0.03	0.04	0.03
	Sex			−1.02	0.58	−0.08
	Position			0.04	0.41	0.00
	Grade level taught			−3.37	0.59	−0.25^***^
	Qualification			0.47	0.84	0.02
**Step 2**		0.35^***^	0.29^***^			
	SPC			0.25	0.13	0.13^*^
	SSC			0.22	0.12	0.11
	CPCa			−0.15	0.10	−0.08
	CPCb			0.21	0.13	0.11
	CSCa			−0.15	0.09	−0.09
	CSCb			0.04	0.11	0.02
	OG			0.37	0.11	0.19^**^
	OT			0.35	0.14	0.18^**^

* p < 0.05;

** p < 0.01;

*** p < 0.001.

SPC, selective primary control; SSC, selective secondary control; CPCa, compensatory primary control (Type a); CPCb, compensatory primary control (Type b); CSCa, compensatory secondary control (Type a); CSCb, compensatory secondary control (Type b); OG, general optimization; OT, optimization in the domain of teaching.

The results show that demographic variables explained 6% of the variance in the Subjective Vitality score [*R*^2^ = 0.06, *F*_(5, 534)_ = 6.97, *p* < 0.001]. Among the control strategy and optimization variables, SPC, OG, and OT were found to be significant predictors of the Subjective Vitality score after controlling for demographic variables (βs = 0.13, 0.19, and 0.18, respectively, *p*s < 0.05). Together, these predictors explained an additional 29% of the variance in the Subjective Vitality score beyond the demographic predictors [Δ*R*^2^ = 0.29, Δ*F*_(8, 526)_ = 28.65, *p* < 0.001]. These results suggest that teachers who employ selective primary control and optimization (in the general and teaching domains) more frequently experience more subjective vitality, and such results lend support to the thesis concerning the primacy of primary control with respect to its adaptive values.

To summarize the results of the abovementioned hierarchical multiple regression analyses, demographic variables explained a relatively small percentage of the variance in the scores that reflected subjective well-being, whereas the control strategy variables explained a comparatively larger percentage of the variance. This finding is in accordance with decades of research on subjective well-being (see Diener et al., 1999). It is worth noting that the significant predictors varied with respect to different facets of subjective well-being. Whereas selective primary control and optimization had benefits in terms of subjective vitality and positive affect, compensatory secondary control and selective secondary control showed adaptive values in the form of satisfaction with one's teaching life. Both the primary and secondary control strivings have adaptive values or benefits. The proposition about the primacy of primary control was only partially supported.

### Life-span trajectories in the endorsement of primary and secondary control strategies

To address the research question about life-span trajectories in the endorsement of primary and secondary control strategies, One-Way ANOVAs were conducted. This analysis allowed us to examine whether there were any statistically significant differences in the OPST subscale scores between the Chinese teachers in different age groups. The teacher sample was classified into three age groups: G1 (20–30 years old; early adulthood, *n* = 230), G2 (31–40 years old; young adulthood, *n* = 248), and G3 (41–60 years old; adulthood after midlife transition, *n* = 159).

The results of the One-Way ANOVAs indicated no significant differences between the Chinese teachers in the three age groups for any of the OPST subscales reflecting primary control except CPCa [*F*_(2, 634)_ = 6.82, *p* = 0.001, η^2^ = 0.02]. Bonferroni-corrected *post-hoc* comparisons further indicated that the difference in CPCa mainly emerged between G1 and G3 (*p* = 0.002) as well as between G2 and G3 (*p* = 0.001). The first two age groups did not differ substantially from each other (*p* = 0.83). The mean CPCa score of G3 was significantly lower than those attained by G1 and G2 (G1: *M* per item = 3.91, *SD* = 4.01; G2: *M* per item = 3.93, *SD* = 3.67; G3: *M* per item = 3.65, *SD* = 4.31). This result suggests that the oldest group was less inclined to seek help. Such a result does not lend full support to the prediction that striving for primary control is stable across adulthood. In terms of the OPST subscales of secondary control, the only subscale that revealed a significant difference between the three age groups was SSC [*F*_(2, 634)_ = 3.50, *p* = 0.03, η^2^ = 0.01]. Bonferroni-corrected *post-hoc* comparisons showed that the G2 teachers scored significantly higher than the G1 teachers, *p* = 0.03, whereas the differences between G1 and G3 (*p* = 1.00) and G2 and G3 (*p* = 0.27) were not significant (G1: *M* per item = 3.72, *SD* = 3.67; G2: *M* per item = 3.89, *SD* = 3.29; G3: *M* per item = 3.76, *SD* = 3.87). This result suggests that the teachers in young adulthood endorsed selective secondary control more frequently than their younger and older colleagues.

**Figure 1:**
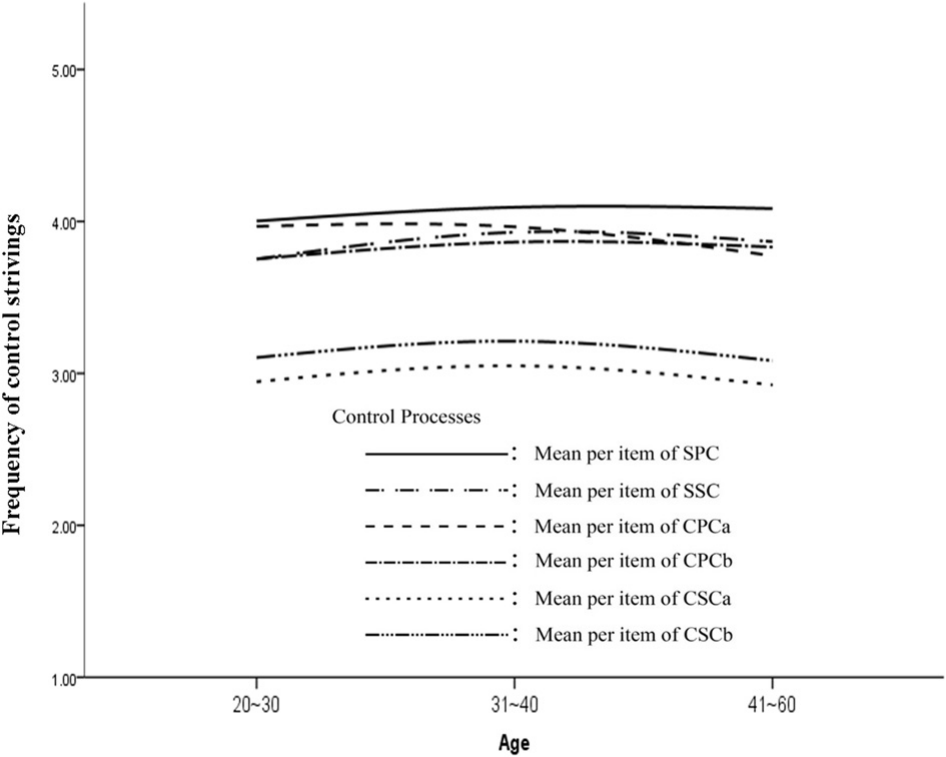
**The life-span trajectories of control strivings among the Chinese teachers**. SPC, selective primary control; SSC, selective secondary control; CPCa, compensatory primary control (Type a); CPCb, compensatory primary control (Type b); CSCa, compensatory secondary control (Type a); CSCb, compensatory secondary control (Type b); OG, general optimization; OT, optimization in the domain of teaching.

To visualize the life-span trajectories of the control strivings, the mean scores on the OPST subscales were plotted on a graph. In Figure 1, which illustrates a cross-sectional comparison, we observe that the endorsement of SPC and CPCb was relatively stable across adulthood. The trend in applying CPCa (i.e., readiness to seek help) appears to be a descending one, indicating that older teachers sought help less frequently. With regard to secondary control, there is an ascending slope in the use of selective secondary control from early adulthood (20–30 years old) to young adulthood (31–40 years old). From young adulthood to adulthood after the midlife transition (41–60 years old), there is a gently descending slope in the endorsement of compensatory secondary control, which is characterized by goal disengagement or goal adjustment, even though the difference in endorsing this type of control striving between the age groups does not reach a statistically significant level. In summary, the life-span trajectories of the control strategies employed by Chinese teachers indicated more divergences from the propositions offered by the motivational theory of life-span control than convergences with these propositions. Nonetheless, caution should be taken in the interpretation of these results due to the limitation of the cross-sectional approach in data collection.

## General discussion

### Convergences with and divergences from the key propositions of the motivational theory of life-span development

The present study aimed to empirically scrutinize the following three key propositions of the motivational theory of life-span development: (1) primary control striving is preferred; (2) primary control striving has benefits; and (3) primary control striving is stable and secondary control striving increases across adulthood (Heckhausen et al., 2010, p. 42). It is evident that the findings from the present study converged with these three key propositions in certain ways and diverged from them in other ways.

With regard to the first fundamental proposition, we found that Chinese teachers clearly preferred using selective primary control and compensatory primary control over compensatory secondary control. Nonetheless, we should note that selective secondary control was also frequently endorsed. In terms of the benefits or adaptive values of control strategies, the results of a series of hierarchical multiple regression analyses showed that different facets of subjective well-being might have different predictors. For example, selective primary control was a significant predictor of subjective vitality and positive affect, whereas selective secondary control and a subtype of compensatory secondary control (goal disengagement or readjustment) were significant predictors of satisfaction with one's teaching life. The relationship between the two subtypes of compensatory primary control and positive affect is noteworthy. CPCb, which is characterized by trying new or more efficient ways to overcome setbacks in teaching practices, was a significant predictor of positive affect. CPCa, which is characterized by a readiness to seek help, did not show any adaptive value. In fact, this type of primary control was a significant negative predictor of positive affect. Taken together, it is questionable whether the primacy of primary control thesis is valid among Chinese teachers.

In terms of the proposition concerning the life-span trajectories of primary and secondary control, the results of the present study do not lend full support to the prediction that primary control striving is stable in adulthood. We found significant differences in the mean scores on the CPCa subscale between Chinese teachers in different age groups. Specifically, the mean scores on the CPCa showed a descending trend throughout adulthood, indicating less of an inclination to seek help among the oldest Chinese teachers. Taken together, the plotted trends for all types of secondary control strategies demonstrated very limited support for the prediction that secondary control striving increases across adulthood. Empirical findings only indicated that teachers in their young adulthood endorsed SSC more frequently than teachers in their early adulthood.

### Incorporating lakatos' ideas about sophisticated falsification into theoretical development

To summarize the results, there were both convergences with the theoretical claims of the motivational theory of life-span development and divergences from these claims. When discussing the dialectical relationship between theory and empirical results, along with deliberating over the development of further research about control processes, it is worthwhile to incorporate Lakatos' ideas about sophisticated falsification (Lakatos, 1970/1999, 1973/1999). Lakatos suggested using a negative heuristic to identify the “hard core” of a research program while using a positive heuristic to articulate or invent auxiliary hypotheses. More specifically, the “hard core” should be immune to refutation within a certain range of time, whereas the auxiliary hypotheses, which serve as a protective belt of the theory, should be subject to readjustment or even replacement. This methodology originates from the recognition that “theories are born in an ocean of anomalies and inconsistencies” (Lakatos, 1973/1999, p. 95). According to Lakatos, these anomalies and inconsistencies should not act as obstacles to genuine scientific developments such as the emergence of novel facts, novel auxiliary theories, and progressive problem-shifts. Despite Lakatos' emphasis on heuristic power and the bracketing of anomalies, he continuously highlighted the need to acknowledge inconsistencies and the state of degeneration of a theory.

What is the hard core of the motivational theory of life-span development? The 15 propositions formulated by Heckhausen et al. (2010) are not of equal theoretical importance. With the goal of advancing the theory in the spirit of Lakatos, it is desirable to specify the hard core from the pool of the 15 propositions. The following three propositions are tentatively suggested: (1) the primacy of primary control, (2) the hypothesized differences in control processes across age groups, and (3) the theoretical formulation around a deadline or a condition of diminishing opportunities. The present study empirically scrutinized the first two aspects. After completing the study, we are in a position to articulate three new auxiliary hypotheses. First, selective secondary control is preferred among professionals. Second, selective secondary control and compensatory secondary control have adaptive values in the form of satisfaction with one's professional life. Third, among professionals, compensatory primary control that is characterized by a readiness to seek help decreases with age. These new auxiliary hypothese call for further empirical scrutiny.

### Integrating the insights of higgins' parenting metaphor into theory development

When deliberating the processes and dynamics of theory development, Higgins' (2004) parenting metaphor is thought provoking. In the early stages of development, a theory needs substantial attention and care from its creator, similar to a good parent-child relationship. Just as good parents do not neglect their children when they are facing problems, good theorists do not give up their intellectual newborns prematurely without defending them. In later stages, it is essential for parents and theorists to allow others to participate in the continued development of their “children.” When reviewing the construction of the motivational theory of life-span development, it is evident that Heckhausen and her associates have played the role of caring parents in the initial stage by implementing a research program and defending the propositions of the theory. In their review paper 2010, Heckhausen et al. displayed an open attitude, inviting psychologists to join the extensive research effort to examine the propositions of the theory. All of the aforementioned endeavors are positive signs of theory development. The next critical trial will be the sensitivity and rigor with which the inconsistencies that emerge from empirical studies are tackled. We suggest that incorporating Lakatos' ideas concerning sophisticated falsification of theoretical developments is desirable because this strategy could ultimately support the development of the theory. When he summarized his parenting metaphor for understanding theory development, Higgins (2004) referred to spoiling, neglecting, abusing, and overprotecting as maladaptive forms of parenting, whereas bolstering and prudence were considered adaptive forms. These insightful ideas also serve as valuable reminders for further endeavor in refining the motivational theory of life-span development.

### Limitations of the present study and suggestions for further studies

Despite the fact that the present study involved Chinese teachers (*N* = 637) from secondary schools of average academic standards from two provinces in the southern and northern parts of mainland China, we cannot generalize the results to the general population of Chinese teachers or to Asian adults. This limitation should be kept in mind when discussing the implications of the present study for the debate about cultural differences with regard to the primacy of primary or secondary control. Another limitation of this study lies in the fact that the comparison of different age groups was based on cross-sectional, rather than longitudinal, data.

In the present sample, we found that Chinese teachers frequently applied selective secondary control, and using this strategy significantly predicted satisfaction with their teaching lives. Furthermore, a subtype of compensatory secondary control that is characterized by goal disengagement or readjustment also significantly predicted satisfaction with one's teaching life. Taken together, these results lend some support to the frequent application of secondary control and its adaptive values in an Asian context, thus serving to shake the foundation of the primacy of primary control. We cannot arrive at a conclusion about the primacy of secondary control because selective primary control was also used extensively and exhibited significant predictive power for subjective vitality and positive affect. To address the longstanding debate about cultural differences in control processes, we offer a more refined pattern rather than a simplified version with the primacy of either primary or secondary control. However, we still cannot explain whether our results are mainly influenced by the teaching domain or the Asian cultural context. To address the debate on cultural differences in a more thorough way, we need to develop a cross-cultural program of research that incorporates different domains and situations in life. For instance, we could extend the cross-cultural investigation into other professions or individuals who experience job loss, retirement, chronic disease, or disability.

The present study aimed to provide empirical scrutiny for the motivational theory of life-span development using a newly constructed domain-specific instrument (OPST) based on the general OPS-Scales. In future research, we can take steps to enrich the conceptualization of primary and secondary control and then translate the ideas into additional OPS subscales that could be applied to any domain. Two ideas are worth considering. The first idea is the concept of “collective forms of primary control” proposed by Morling and Evered (2006). They are insightful in suggesting that primary control may be enacted collectively. The second idea, which entails “deriving a sense of meaning from current experiences,” falls under the category of compensatory secondary control. It is interesting to note that Schulz et al. (1991) mentioned this dimension when discussing the conceptualization of Weisz and his colleagues (Weisz, 1983; Weisz et al., 1984), but it was not incorporated into the general OPS-Scales. Innovating the conceptualization and measurement of key constructs could be regarded as a loosening phase within theory development, whereas empirical scrutiny indicates a tightening phase (see also Fiedler, 2004). Regardless of how rigorously an instrument is developed in terms of control strategies, one should not forget that the data only consist of a single response at one specific time point. To capture the control processes in a more dynamic way, an alternative technique, such as the Experience Sampling Method integrated with convenient e-channels, should be explored. Investigating control processes in the context of microdevelopmental studies constitutes a further alternative approach.

## Conclusions

When the motivational theory of life-span development was given empirical scrutiny in the setting of mainland China with Chinese teachers, evidence that supported and conflicted with the key propositions was found. Striving for different subtypes of primary and secondary control predicted different facets of subjective well-being, thereby preventing a clear conclusion about the primacy of primary or secondary control. The proposition about the life-trajectories of primary and secondary control only gained limited support. To advance the theory, we propose incorporating Lakatos' ideas on sophisticated falsification by specifying the hard core of the motivational theory of life-span development and articulating new auxiliary hypotheses. We also suggest enriching the conceptualization of primary and secondary control and trying innovative methods that capture the dynamic nature of control processes. All of these endeavors will have practical implications for promoting well-being and optimal functioning.

### Conflict of interest statement

The authors declare that the research was conducted in the absence of any commercial or financial relationships that could be construed as a potential conflict of interest.
